# Glucose-fed microbiota alters *C. elegans* intestinal epithelium and increases susceptibility to multiple bacterial pathogens

**DOI:** 10.1038/s41598-024-63514-w

**Published:** 2024-06-07

**Authors:** Samuel F. Kingsley, Yonghak Seo, Alicia Wood, Khursheed A. Wani, Xavier Gonzalez, Javier Irazoqui, Steven E. Finkel, Heidi A. Tissenbaum

**Affiliations:** 1 Department of Molecular, Cell and Cancer Biology, UMass Chan Medical School, Worcester, MA 01605 USA; 2 Department of Microbiology and Physiological Systems, UMass Chan Medical School, Worcester, MA 01605 USA; 3 Molecular and Computational Biology Section, Department of Biological Sciences, University of Southern California, Los Angeles, CA 90089-2910 USA; 4 Program in Molecular Medicine, UMass Chan Medical School, Worcester, MA 01605 USA

**Keywords:** Genetics, Microbiology, Physiology, Diseases, Endocrinology, Molecular medicine, Risk factors

## Abstract

Overconsumption of dietary sugar can lead to many negative health effects including the development of Type 2 diabetes, metabolic syndrome, cardiovascular disease, and neurodegenerative disorders. Recently, the human intestinal microbiota, strongly associated with our overall health, has also been known to be affected by diet. However, mechanistic insight into the importance of the human intestinal microbiota and the effects of chronic sugar ingestion has not been possible largely due to the complexity of the human microbiome which contains hundreds of types of organisms. Here, we use an interspecies *C. elegans*/*E. coli* system, where *E. coli* are subjected to high sugar, then consumed by the bacterivore host *C. elegans* to become the microbiota. This glucose-fed microbiota results in a significant lifespan reduction accompanied by reduced healthspan (locomotion), reduced stress resistance, and changes in behavior and feeding. Lifespan reduction is also accompanied by two potential major contributors: increased intestinal bacterial density and increased concentration of reactive oxygen species. The glucose-fed microbiota accelerated the age-related development of intestinal cell permeability, intestinal distention, and dysregulation of immune effectors. Ultimately, the changes in the intestinal epithelium due to aging with the glucose-fed microbiota results in increased susceptibility to multiple bacterial pathogens. Taken together, our data reveal that chronic ingestion of sugar, such as a Western diet, has profound health effects on the host due to changes in the microbiota and may contribute to the current increased incidence of ailments including inflammatory bowel diseases as well as multiple age-related diseases.

## Introduction

Improper regulation of sugars, such as glucose, can lead to the development of type 2 diabetes, obesity, inflammatory bowel diseases, cardiovascular disease, and neurodegenerative diseases which are estimated to affect 10–20% of the worldwide population. The numbers of those afflicted are rising in large part due to the increased amounts of dietary sugar found most commonly in the Western diet^1^.

Importantly, the human intestinal microbiota has been shown to be affected by diet which then influences overall health. In fact, studies suggest that a high-sugar diet can alter the gut microbiota, leading to age-associated illness^2,3^. However, mechanistic insight into the critical role of the human intestinal microbiota and the effects of chronic sugar ingestion has not been possible due to the complexity of the human microbiome which contains hundreds of types of organisms. Additionally, much of the data for human studies comes from fecal samples, representing only a small portion of the microbes lining the large intestine.

Here, we use an interspecies *Caenorhabditis elegans (C. elegans)/Escherichia coli* (*E. coli*) research platform to simplify direct assessment of the importance of the microbiota, how it is influenced by a high sugar diet, and how this diet impacts the host. As bacterivores, *C. elegans* have an obligatory symbiotic relationship with microbes as their food source. Interestingly, for normal laboratory growth, *C. elegans* only require one bacterial strain. Therefore, this system allows for univariable analysis of both the intestinal microbiota and the host.

Addition of glucose to the *C. elegans* bacterial diet results in changes in lifespan and healthspan^4–8^. These studies added glucose either directly into the agar or on top of the agar plate. Therefore, the additional glucose is in contact with both bacteria and *C. elegans*, resulting in decreased lifespan, reduced healthspan (locomotion), reduced fecundity, and changes in fat storage^4–8^.

To separate the effects of glucose on *C. elegans* and bacteria, previously^9^, we developed an experimental procedure where *E. coli* is incubated with glucose then heat-killed, which results in a glucose-fed bacterial diet^9^. Here, to better mimic the mammalian intestine exposed to a high sugar diet, we pre-treat the bacteria with glucose, but rather use live bacteria as the food source. The live bacteria inhabit *C. elegan*s and become the intestinal microbiota, producing a glucose-fed microbiota. Our data reveal that the glucose-fed microbiota results in a significant decrease in lifespan as well as a reduction in multiple healthspan parameters including locomotion, stress resistance, and changes in behavior and feeding. We also find that the glucose-fed microbiota results in increased intestinal bacterial density, increased concentration of reactive oxygen species, promoted dysregulation of immune effectors, accelerated age-related development of intestinal cell permeability, increased intestinal distention, and increased susceptibility to multiple bacterial pathogens. Taken together, we believe *C. elegans* with a glucose-fed microbiota mimics the microbiota of a person on a Western high added sugar diet.

## Results and discussion

**Figure 1:**
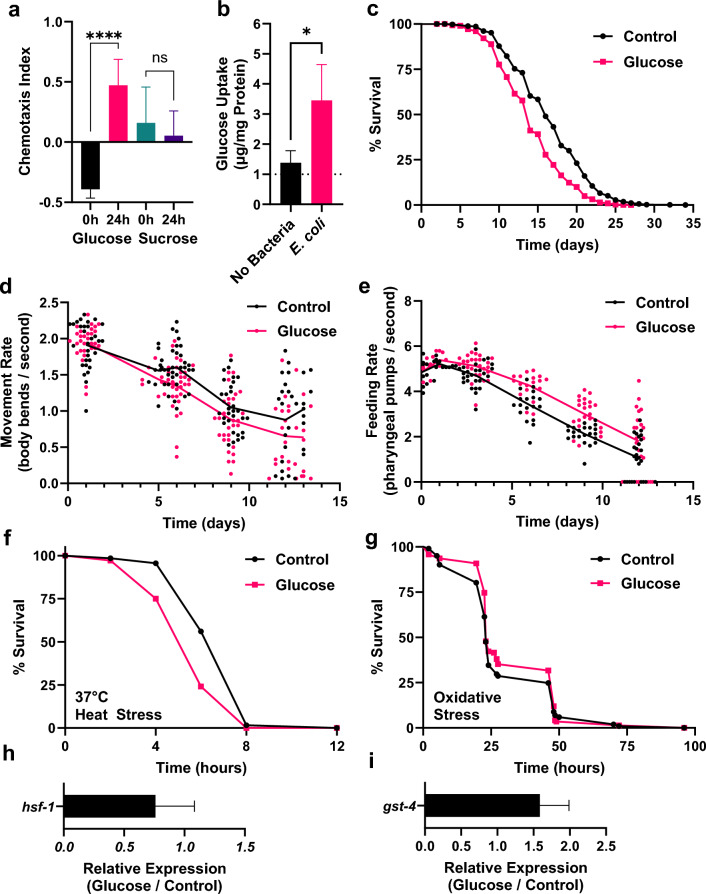
Bacterial incubation with Glucose is required for changes in behavior, lifespan, and health consequences. (**a**) Chemotaxis Index of *C. elegans* to glucose or sucrose incubated with *E. coli* for 0 or 24 h (left to right, mean ± s.d. (n = 344, 278, 264, 327 animals respectively, L-R), Glucose ****p< 0.001.Sucrose not significant (ns) unpaired t-test). (**b**) Whole animal glucose uptake of *C. elegans* after growing 2 days either on 2% glucose or 0% control plates without bacteria or 2% glucose versus 0% control plates seeded with *E. coli* mean ± s.d. (n = 600 animals per treatment), *p < 0.05 by unpaired t-test). (**c**) Lifespan assay of *C. elegans* with glucose-fed HT115 *E. coli* (Control mean ± s.d. = 16.5 ± 4.82 days (n = 1028), Glucose mean ± s.d. = 14.2 ± 4.27 days (n = 1049, ****p < 0.001 by Log-rank (Mantel–Cox) test). (**d**) Healthspan-movement in liquid of *C. elegans* growing with a control or glucose-fed microbiota over time (Control n = 129, Glucose n = 127), *p < 0.05 by unpaired t-test at day 6). (**e**) Feeding rate as measured by pharyngeal pumping of *C. elegans* growing with either a control or glucose-fed microbiota (Control n = 118, Glucose n = 118), **p < 0.01 at day 3 ***p < 0.001 at day 6 ****p < 0.0001 day 9 by unpaired t-test). (**f**) Resistance to heat stress of *C. elegans* grown for 6 days with either a control or glucose-fed microbiota (Control mean ± s.d. = 0.26 ± 0.06 days (n = 347), Glucose mean ± s.d. = 0.21 ± 0.03 days (n = 344), **p < 0.01 by Log-rank (Mantel–Cox) test). (**g**) Resistance to oxidative stress of *C. elegans* grown for 6 days post L4 with a control or glucose-fed microbiota (Control mean ± s.d = 1.21 ± 0.67 (n = 101) days, Glucose mean ± s.d. = 1.3 ± 0.66 (n = 142) days, *p* > 0.05, Log-rank (Mantel–Cox test). (**h**, **i**) Relative RT-qPCR expression of *hsf-1* and *gst-4* in *C. elegans* grown with either a control or glucose-fed microbiota for 6 days post L4. mean ± s.d. (n = 500).

Previously^9^, our data revealed that bacterial processing of added dietary glucose was required for the negative physiological response of the host. To expand these results, we characterized the sensory response of *C. elegans* and performed chemotaxis assays (Fig. 1a). Initially, animals avoid glucose, however, after 24 h of incubation with *E. coli,* glucose becomes significantly attractive (*p* < 0.05, Fig. 1a). Testing more frequently revealed that the chemotaxis response to glucose is progressive and time-dependent; (Supplemental Fig. 1a). In a similar experiment, where sucrose was added to the bacteria, no significant change was observed at either time point (*p* > 0.05, Fig. 1a). This is an important control as *E. coli* do not metabolize sucrose^10^. Therefore, our data illustrate the importance of documenting the time *E. coli* are in contact with glucose prior to addition of *C. elegans* for future studies with an added-glucose/high-glucose bacterial diet.

Since bacterial processing of glucose affects the attraction of *C. elegans* to *E. coli,* we questioned whether glucose uptake would also be altered. Therefore, next we measured glucose levels of animals growing for 2 days with or without bacteria on agar plates containing either 0% or 2% added glucose. As shown in Fig. 1b, without bacteria, *C. elegans* have a 38% increase in glucose levels when comparing growth on agar plates containing either 0% or 2% added glucose. However, in contrast, animals growing on agar plates with *E. coli* have a significantly greater increase in internal glucose of 345% when comparing growth on 0% with 2% added glucose (*p* < 0.05, Fig. 1b). These results further indicate a role for the bacteria in the animals’ ability to uptake glucose.

Next, to better mimic the mammalian intestine under the duress of a Western high sugar diet, we modified the protocol^9^, such that *E. coli* are exposed to glucose for three days and live, glucose-grown bacteria serve as the diet for the host *C. elegans*. The live bacteria will inhabit *C. elegan*s and become the intestinal microbiota, resulting in a glucose-fed microbiota. Consistently, we observed that the glucose-fed microbiota significantly reduced *C. elegans* lifespan from control 16.5 ± 4.8 days (n = 1028) to 14.2 ± 4.3 days (n = 1049) for animals with glucose-fed microbiota (*p* < 0.01, Fig. 1c). Increased amounts of glucose resulted in further shortening of lifespan (Supplemental Fig. 1b). From these results, we chose 0.8% glucose for the rest of the experiments.

We tested the effect of glucose on multiple non-pathogenic *E. coli* strains. As shown in Supplemental Fig. 2a–c, using 3 additional *E. coli* strains, consumption of live glucose-fed *E. coli* OP50, *E. coli* HB101, or *E. coli* BW25113 resulted in a significant decrease in lifespan (*p* < 0.01). Statistical analysis of the lifespan experiments can be found in Supplementary Table S1. Given that the response to glucose was universal, the remainder of the experiments utilized *E. coli* strain HT115, commonly used in *C. elegans* experiments.

**Figure 2:**
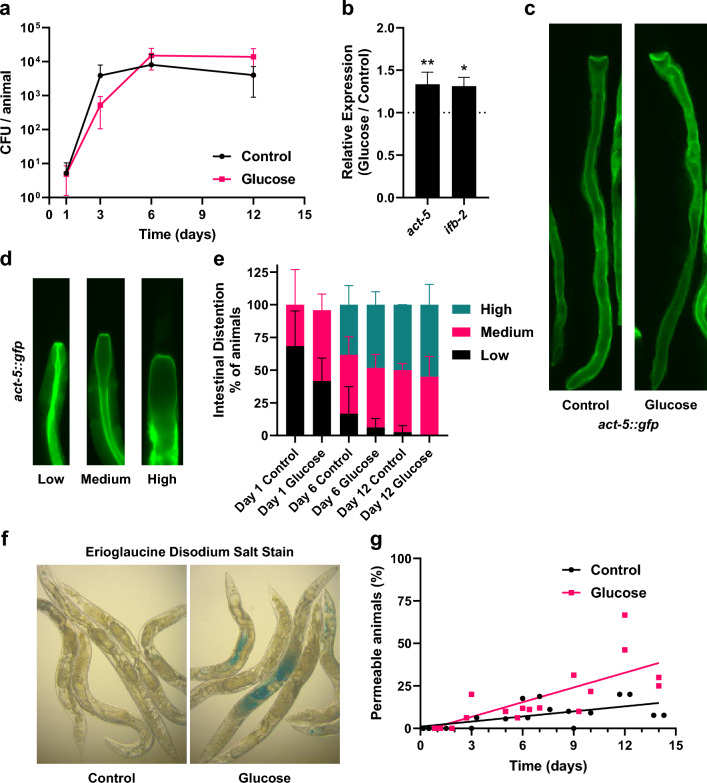
Glucosefed microbiota promotes intestinal bacterial growth, intestinal distention, and intestinal permeability. (**a**) Bacterial density of *E. coli* inside *C. elegans* after growing with a control or glucose-fed microbiota for 1–12 days mean ± s.d. (n = 550 per experimental group), *p < 0.05 at day 12 by unpaired t-test. (**b**) Relative RT-qPCR expression of *act-5* and *ifb-2* in *C. elegans* with a control or glucose-fed microbiota grown for 6 days post L4, mean ± s.d. (n = 400 per experimental group), *act-5* **p < 0.01, *ifb-2* *p < 0.05. (**c**) Photos of *act-5::gfp* growing with control or glucose-fed microbiota for 12 days post L4. (**d**) Examples of *act-5::gfp* with either low, medium, or high levels of intestinal distention. (**e**) Quantification of *act-5::gfp* intestinal distention after growing with either a control or glucose-fed microbiota for 1, 6, or 12 days post L4, mean ± s.d. (control n = 131 and glucose-fed microbiota n = 121), distention phenotype percentages averaged across 3 biological replicates). (**f**) Photograph of “Smurf” staining with erioglaucine disodium salt dye within *C. elegans* aged with either control or glucose-fed microbiota for 12 days post L4. (**g**) Quantification of “Smurf” staining in the body cavity of *C. elegans* growing with either a control or glucose-fed microbiota for 0–14 days, linear regression line fit to data to show trend (control n = 283 and glucose-fed microbiota n = 281), each dot represents mean staining of 10–20 animals; *p < 0.05 at day 9, ****p < 0.001 at day 12, **p < 0.01 at day 14 by unpaired t-test.

To further evaluate how the aging process was affected by a glucose-fed microbiota, next we examined the animals’ healthspan. As shown in Fig. 1c, healthspan, as measured by locomotion/movement in liquid (also termed body bends/swimming/thrashing), was significantly reduced (*p* < 0.01) with a glucose-fed microbiota at 6 days of age and remained reduced at all ages The decreased lifespan and reduction in movement was not at the same magnitude as animals consuming a high-glucose diet^11^. This may be due to the particular concentration of glucose metabolized by the bacteria. Here, we used a fraction of that seen in other studies. Aging animals with a glucose-fed microbiota revealed that at days 3, 6, and 9, there was a significant increase in the feeding rate measured by pharyngeal pumping^12,13^ (*p* < 0.05, Fig. 1e). The attraction to sugary food is unlikely to be the reason for increased pumping since, as our data shows, this is a delayed response (Fig. 1a,e), Therefore, examining both healthspan and lifespan showed immediate and long-term consequences of the glucose-fed microbiota.

Concurrently, while scoring animal survival with a glucose-fed microbiota, the avoidance of the bacterial lawn was also scored as animals tend to avoid xenobiotic or pathogenic bacteria^14^. Animals consistently showed less avoidance of the glucose-fed bacteria and remained on the bacterial lawn for longer periods of time relative to the control animals with a no-added-glucose diet (Supplemental Fig. 2d).

We then tested the ability of animals aging with a glucose-fed microbiota to maintain homeostasis, another health assessment^15^. Animals aged 6 days with a glucose-fed microbiota show a significant, 25% reduction in mean heat stress survival (*p* < 0.05; Fig. 1f), and no significant change in mean oxidative stress survival with paraquat (*p* > 0.05; Fig. 1g). Interestingly, RT-qPCR of animals aged for 6 days with the glucose-fed microbiota showed reduced expression of a key transcription factor for thermotolerance, heat shock factor-1, *hsf-*1^16,17^ (Fig. 1h) and increased expression of *gst-4* (Fig. 1i), glutathione-S-transferase-4 (*gst-4*)^18^, a marker for oxidative stress. One possibility, in agreement with these results, is that animals with a glucose-fed microbiota have reduced heat resistance due to suppression of *hsf-1* gene expression. Accordingly, the increased gene expression of *gst-4* would allow for no change in oxidative stress resistance.

Animals with a glucose-fed microbiota show a decrease in lifespan as well as an increase in the feeding rate (as mentioned by pharyngeal pumping). Previously, a connection between lifespan, food intake, and the intestinal microbes was observed such that lifespan is extended on killed bacteria^19^. Moreover, bacterial overgrowth has been shown to be a cause of death in *C. elegans*^19^. To confirm that the bacteria are colonizing the intestine, we examined growth of bacteria within the aging *C. elegans* intestine using a fluorescently tagged *E. coli* strain, OP50-GFP. When fed OP50-GFP *E. coli* as *C. elegans* aged, GFP accumulates significantly within the intestine (*p* < 0.05, Supplemental Fig. 3a,b). We then isolated and quantified the bacteria residing within the *C. elegans* intestine measuring the colony forming units (CFU). As a function of age, intestinal bacterial density increases similarly to the fluorescence quantification (Supplemental Fig. 3c).

**Figure 3:**
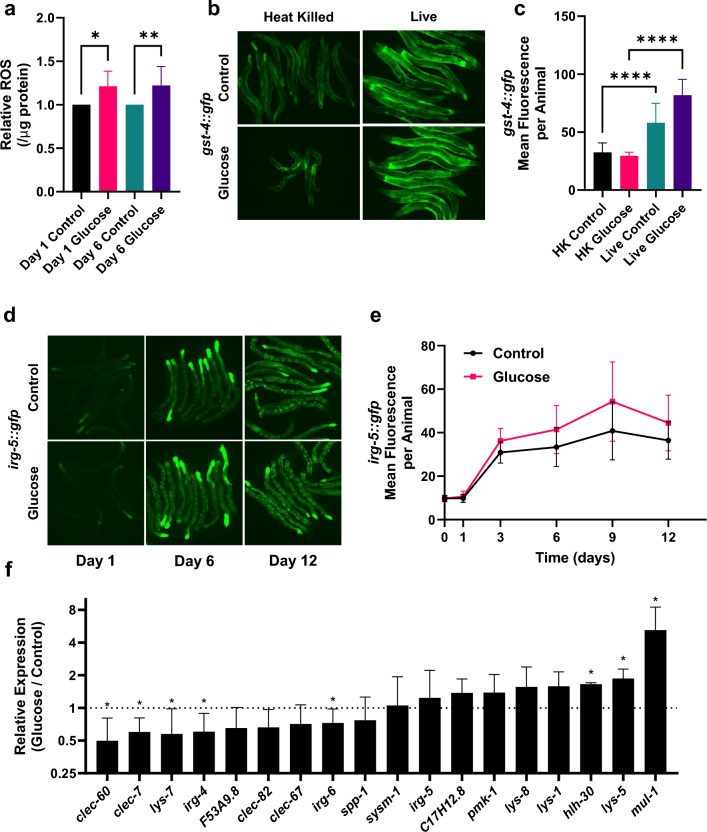
A glucose-fed microbiota promotes oxidative stress. (**a**) Reactive Oxygen Species (ROS) of *C. elegans* growing with either a control or glucose-fed microbiota for 1 or 6 days post L4, displayed as the amount of ROS relative to age matched samples (mean ± s.d., (n = 300 per column) *p < 0.05 at day 1, **p < 0.01 at day 6 by unpaired t-test). (**b**) Photographs of *gst-4::gfp* animals after growing with either a heat killed or live control and either a control or a glucose-fed microbiota for 6 days post L4. (**c**) Quantification of *gst-4::gfp* whole body fluorescence after growing with either a heat killed (HK) or live control and glucose-fed microbiota for 6 days post L4mean ± s.d., (HK control microbiota n = 31, live control microbiota n = 22 , HK glucose-fed microbiota n = 35, and live glucose-fed microbiota n = 32, ****p < 0.001 by unpaired t-test). (**d**) Photographs of *irg-5::gfp* after growing with a control or glucose-fed microbiota for 1, 6, or 12 days. (**e**) Quantification of *irg-5::gfp* whole body fluorescence after growing with either a control or glucose-fed microbiota for 1–12 days post L4, mean ± s.d. (n = 155 control microbiota, n = 161 glucose-fed microbiota, **p < 0.01 at day 6, ****p < 0.001 at day 9, *p < 0.05 at day 12 by unpaired t-test). (**f**) Relative RT-qPCR expression of a panel of innate immune reactive genes. *C. elegans* grown with either a control or glucose-fed microbiota for 6 days posst L4 (mean ± s.d. (n = 400 per experimental group), *p < 0.05 for *clec-60*, *clec-7*, *lys-7*, *irg-4*, *irg-6*, *hlh-30*, *lys-5*, *mul-1* by unpaired t-test).

To ensure that bacterial proliferation was not linked to the GFP tag, we examined animals with a glucose-fed microbiota consuming *E. coli* HT115. As shown in Fig. 2a, intestinal CFU increased at 6 days and significantly increased at 12 days (*p* < 0.05). Therefore, these data show that *C. elegans* exhibit intestinal bacterial packing/overgrowth within the animal as a function of both diet and age. Interestingly, there was no correlation between the mean pumping rate and intestinal bacterial density as the animals aged showing that the increased CFU was not due to increased consumption (Supplemental Fig. 3d).

Previously, *hsf-1,* has been shown to regulate both lifespan and thermotolerance^16,20^ similar to animals with a glucose-fed microbiota. *hsf-1* also modifies the actin cytoskeleton^16,21,22^. Therefore, we next questioned whether the glucose-fed microbiota affected the actin cytoskeleton and its function in maintenance of intestinal epithelium integrity. We first examined two genes that function to maintain the intestinal epithelium barrier, the actin *act-5*, and the intermediate filament proteins b, *ifb-2. act-5* functions as the primary actin filament within the intestinal microvilli^23^, with *ifb-2* as an intermediate filament protein which functions to anchor *act-5* on the apical border of the intestinal epithelium^24,25^. RT-qPCR of day 6 animals with a glucose-fed microbiota reveals both *act-5* and *ifb-2* were significantly upregulated (*p* < 0.05, Fig. 2b). In a second series of experiments, we examined *act-5::gfp* transgenic animals aged with and without a glucose-fed microbiota (Fig. 2c). Quantification showed no significant changes in *act-5::gfp* whole body fluorescence intensity as the animals aged (*p* > 0.05, Supplemental Fig. 4). However, as animals aged, overall fluorescence steadily declined from day 3 to day 12, indicating a change in the actin cytoskeleton in older animals consistent with previous studies^26^ (Supplemental Fig. 4).

**Figure 4:**
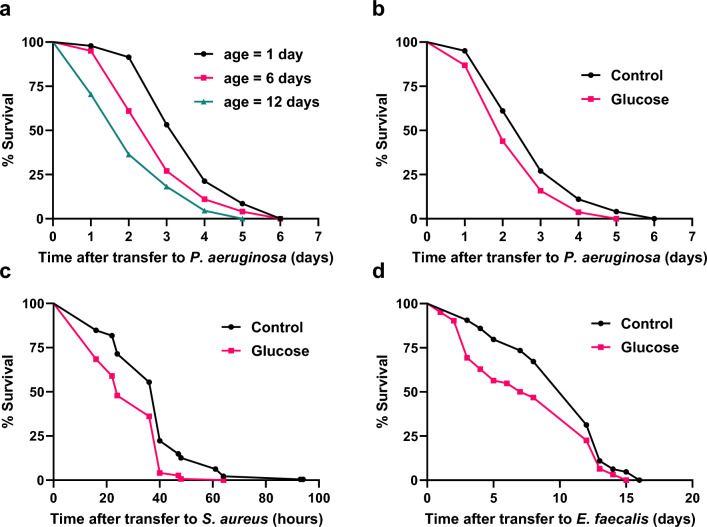
Aging with a glucose-fed microbiota results in susceptibility to multiple bacterial pathogens. (**a**) Survival of *C. elegans* aged 1, 6, or 12 days with a control microbiota before transfer to *P. aeruginosa* (1 day mean ± s.d. = 3.72 ± 1.12 days (n = 47), 6 day mean ± s.d. = 2.98 ± 1.16 days (n = 100), 12 day mean ± s.d. = 2.3 ± 1.17 days (n = 44), **p =  < 0.01 between 1 and 6 days, ****p < 0.01 between 1 and 12 days by Log-rank (Mantel–Cox) test). (**b**) Survival of *C. elegans* on *P. aeruginosa* after growing with either a control or glucose-fed microbiota for 6 days (Control mean ± s.d. = 2.98 ± 1.16 days (n = 100), Glucose , mean ± s.d. = 2.5 ± 0.1 days (n = 107), **p < 0.01 by Log-rank (Mantel–Cox) test). (**c**) Survival of *C. elegans* on *S. aureus* after growing with either a control or glucose-fed microbiota for 6 days post L4 (Control mean ± s.d. = 1.55 ± 0.63 days (n = 230), Glucose mean ± s.d. = 1.2 ± 0.47 days (n = 275) ****p < 0.001 by Log-rank (Mantel–Cox) test). (**d**) Survival of *C. elegans* on *E. faecalis* after growing with a control or glucose-fed microbiota for 6 days (Control mean ± s.d. = 10.3 ± 3.86 days (n = 64), Glucose mean ± s.d. = 7.98 ± 4.7 days (n = 62),*p < 0.05 by Log-rank (Mantel–Cox) test).

Further analysis of *act-5::gfp* expression in animals with and without a glucose-fed microbiota revealed bacterial distention in the upper intestine with age. We classified the bacterial distention of the upper intestine into three levels: low, medium, and high (Fig. 2d). Quantification of animals as they aged revealed that as a function of age, there was greater bacterial distention for animals aging with a glucose-fed microbiota. Additionally, our analysis showed day 12 control animals had a similar profile to day 6 animals with a glucose-fed microbiota, suggesting premature aging (Fig. 2e). Overall, the proportion of animals scored as medium and high levels of bacterial distention was elevated in the animals with a glucose-fed microbiota.

In another series of experiments, we examined the integrity of the intestinal epithelium using the “Smurf” assay, originally used in *Drosophila*^27,28^ and modified successfully for use in *C. elegans*^29^. The Smurf assay is a staining procedure using erioglaucine disodium salt dye which is taken up by permeable membranes, but is excreted by healthy animals, with an example of a positively stained aged animal in Fig. 2f. As expected, young animals showed little permeability in the intestinal barrier, however, at 9 days, the glucose-fed microbiota confers a significant increase in permeability (*p* < 0.05, Fig. 2g). By day 12, on average 56% of animals with the glucose-fed microbiota exhibited a Smurf phenotype compared to 20% with a control microbiota. Further examination of the erioglaucine disodium salt-stained animals revealed that there were dye retention changes in different tissues (pharynx, upper intestine, mid-intestine/vulva, lower intestine). In general, dye retention increased with age in all tissues. Animals with a glucose fed microbiota show little differences at early age but as animals grew older, we observed increased dye retention in the mid intestine, vulva, and lower intestine (Supplemental Fig. 5a–e). Therefore, our data reveal animals with a glucose-fed microbiota have increased intestinal epithelial disruption and intestinal bacterial overgrowth.

In humans, illnesses such as small intestinal bacterial overgrowth (SIBO) are characterized by excessive growth of bacteria in the small intestine^30^. Interestingly, multiple homeostatic systems control the bacterial population in the small intestine, but conditions such as aging and diabetes disrupt these mechanisms and lead to SIBO^30^. Bacterial over-proliferation illnesses such as SIBO leads to inflammation and activation of the immune response. Therefore, next we examined if there was any connection between bacterial overgrowth caused by the glucose-fed microbiota, inflammation, and the immune response.

In *C. elegans*, there is no true inflammation. However, it is well documented that, similar to mammalian phagocytes, *C. elegans* produce reactive oxygen species (ROS) in response to infection^31^. We found whole animal ROS was significantly increased at both days 1 and 6 with a glucose-fed microbiota (*p* < 0.05, Fig. 3a). The increased ROS could emanate from the bacteria within the animal or from the animal itself. To further examine this, we used a well-known marker gene for oxidative stress fused to GFP*, gst-4::gfp* and exposed animals to both live and heat-killed glucose-fed bacteria. As shown in Fig. 3b and quantified in Fig. 3c, animals elicit upregulation of *gst-4* only with live bacteria. Therefore, animals with a live glucose-fed microbiota have an increase in oxidative stress as well as increased ROS. The bacterial overgrowth and increased ROS production when animals have a glucose-fed microbiota may indicate that the animals are perceiving a pathogen, which in *C. elegans* is sensed by the innate immune system. When *C. elegans* are exposed to a bacterial pathogen including *Pseudomonas aeruginosa* (*P. aeruginosa*)*, Staphylococcus aureus *(*S. aureus*), *Yersinia pestis* and *Enterococcus faecalis* (*E. faecalis*), gene expression of immune effectors including CUB-like genes, lectins, lysozymes, and PUFA genes increases^32–35^. To examine the relationship between innate immunity and the glucose-fed microbiota, we first examined a fluorescent transcriptional reporter for an immune response gene, *irg-5*^36–38^ since expression of *irg-5* is dependent on multiple immune pathways^32,35,37,39^. After 6 days, the glucose-fed microbiota significantly increased expression of *irg-5::gfp* (*p* < 0.05, Fig. 3d,e). We then examined innate immune effector genes previously linked to a bacterial pathogenic response including C-type lectins, lysozymes, mucin, MAP kinase, and other infection related/innate immune genes^32–34^. As shown in Fig. 3f, after aging 6 days with or without a glucose fed microbiota, RT-qPCR showed ~ 50% of the innate immune genes tested were significantly changed (*p* < 0.05). Genes significantly downregulated include *clec-60*, *clec-7*, *lys-7*, *irg-4*, and *irg-6*. Genes significantly upregulated include *hlh-30*, *lys-4*, and *mul-1*. Similar to the results with the *irg-5::gfp* reporter, RT-qPCR showed *irg-5* expression was increased. However, though animals with a glucose-fed microbiota showed some significant changes in several innate immune genes, this was not the same magnitude as changes when *C. elegans* undergo a pathogenic response. For example: *irg-5* changes 62-fold with *Yersinia pestis*^32^ and 20-fold with *Pseudomonas aeruginosa* (*P. aeruginosa)*^34^ while *irg-4* changes sixfold with both *Yersinia pestis*^32^ and *P. aeruginosa*^34^. Therefore, animals with a glucose-fed microbiota show signs of a bacterial infection (increased CFU/bacterial overgrowth, increased ROS production and increased oxidative stress)^32–35^. However, together with the gene expression data, we suggest that rather than a bacterial pathogen infection, a glucose-fed microbiota results in dysregulation of immune function.

To further examine the consequences of the immune dysregulation in combination with increased intestinal epithelial permeability, we tested the resistance of animals aging with a glucose-fed microbiota to multiple bacterial pathogens. Animals with a glucose-fed microbiota were aged for either 1, 6, or 12 days*.* As shown in Fig. 4a, as animals age on a control diet, they exhibit an age-dependent decrease in survival to the bacterial pathogen *P. aeruginosa*. Next, we examined the effects of aging combined with a glucose-fed microbiota on bacterial pathogenic tolerance. As shown in Fig. 4b, animals aged for 6 days with a glucose-fed microbiota showed a significant reduction in survival when compared to animals aged on a control diet prior to exposure to the bacterial pathogen *P. aeruginosa* (*p* < 0.05, Fig. 4b). Similarly, as shown in Fig. 4c, animals aged for 6 days with a glucose-fed microbiota showed a significant reduction in survival compared to animals grown on a control diet prior to exposure to the bacterial pathogen, *S. aureus* (*p* < 0.05, Fig. 4c). Additionally, animals with a glucose-fed microbiota also showed markedly reduced resistance when compared to animals grown on a control diet prior to exposure to a third bacterial pathogen, *E. faecalis* (*p* < 0.05, Fig. 4d). Therefore, as animals age, the ability to respond to bacterial pathogens declines. Our data also reveal the importance of a healthy microbiota in bacterial pathogen resistance since across multiple bacterial pathogens, animals with a glucose-fed microbiota show vulnerability and decreased resistance to multiple bacterial pathogenic infections.

In summary, our results reveal that animals with a glucose-fed microbiota show lifelong significant alterations in both lifespan and healthspan. Our data revealed that the health consequences of a glucose-fed microbiota occur progressively. Initially, animals show an increased attraction to their food, mild gut distention, and increased ROS. By day 3, animals show an increase in pharyngeal pumping/feeding rate. By day 6, there is increased bacterial accumulation, increased intestinal distention, increased ROS, increased bacterial pathogen susceptibility, reduced heat stress resistance, reduced locomotion, and immune dysregulation. By day 9, there is increased permeability of the intestinal epithelium. By day 12, animals with a glucose-fed microbiota show an increased chance of death. Interestingly, all phenotypes associated with a glucose-fed microbiota are accelerated by age, occurring days before the mean survival time. This suggests that alleviating these symptoms could change the course of an animal's health and survival.

Overall, our data reveal that two important factors, the diet and the aging process contribute to *C. elegans* lifespan and healthspan. We clearly show that as animals age with a glucose-fed microbiota, they have reduced lifespan and become unhealthy as exemplified by an increased susceptibility to multiple bacterial pathogens. We show the critical importance of a healthy microbiota throughout the aging process. Together, our data show the potential link between diet and bacterial infection and possibly represent a new avenue for therapeutics.

## Methods

### Strain maintenance

All *C. elegans* strains were maintained at 20 °C using standard *C. elegans* techniques^40^. Wild type N2 *C. elegans* was used for most experiments. The following transgenes were used: AY101 *acIs101* [F35E12.5p::GFP + rol-6(su1006)], ERT60 *jyIs13* [*act-5p*::GFP::ACT-5 + *rol-6(su1006*)] II., CL2166 *dvIs19* [(pAF15)*gst-4p*::GFP::NLS] III.

### Chemotaxis assay

The chemotaxis assay was adapted from^41,42^. Standard NGM 60 mm plates were divided into 4 quadrants, with a 10 mm diameter circle in the center, and 4 dots (one in each quadrant 1 mm from the edge of the plate). 10μL of 2 M D-Glucose (G8270-1 KG, Sigma Aldrich) or 2 M Sucrose (S0389-500G, Sigma Aldrich) solubilized in M9 was pipetted onto the dots of two opposing quadrants with control (M9) on the last two quadrants and allowed to dry. Then, 10 μL of OP50 *E. coli* was spotted on top of the dried treatment and plates were either left to incubate at room temperature for 24 h or used immediately after bacteria had dried. Well-fed *C. elegans* adults were picked from stock OP50 *E. coli* plates, washed three times with M9, and picked onto the center of all assay plates. After one hour, plates were placed at 4 °C for 15 min to slow/immobilize animals for ease of scoring and the location of the animals recorded. The chemotaxis index refers to the (# of animals on treatment − # of animals on control)/total # of animals. Experiments were performed on 6 biological replicates with 50 animals each, and statistics calculated using an unpaired t-test with GraphPad Prism 10 (Graphpad Software).

### Glucose-fed microbiota

Stabs of stock frozen cultures of OP50 *E. coli*, HT115(DE3)-L4440 (referred to as HT115 *E. coli*), HB101 *E. coli*, and BW25113 strain *E. coli* were used to inoculate 50 mL LB cultures to start each culture. HT115/L4440 *E. coli* was grown in the presence of ampicillin, HB101 *E. coli* in the presence of streptomycin, and OP50 *E. coli* and BW25113 *E. coli* with no antibiotics. Bacterial cultures were grown in LB with either sterile filtered ddH_2_O (control) or 0.8% D-Glucose aerobically at 37 °C on a shaker plate for 3 days, then pipetted onto NGM plates and allowed to dry for 2 days. *C. elegans* were picked onto plates and aged for analysis. Plates were stored at 4 °C for a maximum of two weeks and brought to room temperature prior to use.

### Measurement of total glucose

Total glucose of *C. elegans* lysates was measured using a Glucose Assay Kit (GAGO20-1KT, Sigma-Aldrich) according to the manufacturers’ protocol. NGM plates with either 0% or 2% added glucose were seeded with/without 250uL of OP50 *E. coli* and then dried for 2 days. Approximately 100 *C. elegans* L4s were picked onto each plate and grown at 20 °C for 2 days. Animals were then picked off plates, washed 2 × with M9 buffer, and then frozen at − 80 °C. Frozen pellets were reconstituted in 7 volumes of RIPA (150 mM NaCl, 50 mM Tris pH 7.4, 1% Triton X-100, 0.1% Sodium Dodecyl Sulfate, 1% Sodium Deoxycholate) buffer freshly mixed with Protease Inhibitor Cocktail (P2714-1BTL, Sigma Aldrich). Samples were then lysed on ice for 30 s total using a Microson XL2000 probe sonicator at 25% power. Each sample was also analyzed with a Pierce Coomassie Plus (Bradford) assay (23,236, Thermo Scientific) and Glucose values were normalized by protein values. Experiments were performed on 3 biological replicates, and statistics were calculated using an unpaired t-test with GraphPad Prism 10 (Graphpad Software).

### Lifespan assay

*C. elegans* L4s were picked onto control or glucose-fed *E. coli* plates and grown at 20 °C (∼25 animals per 60 mm plate). Animals were transferred to new plates every 2 days and scored by gently tapping with a platinum wire pick daily while producing progeny and then every 2–3 days. Animals that did not respond were scored as dead. Animals that were lost, desiccated, bagged, or died from vulva bursting were censored from the analysis. Each figure shows cumulative data collected from 2 or more biological replicates, and statistics were calculated by a Kaplan–Meier survival curve utilizing a Log-rank (Mantel–Cox) test with GraphPad Prism 10 (Graphpad Software) and shown in Supplementary Table 1.

### Locomotion/movement in liquid

Individual animals were picked onto an unseeded NGM plate, then 10μL of M9 buffer was pipetted onto each animal, and the number of body bends was counted over 30 s. Experiments were performed on 3 biological replicates with approximately 10 animals each, and statistics were calculated by an unpaired t-test using GraphPad Prism 10 (GraphPad Software).

### Pharyngeal pumping/feeding rate

*C. elegans* were counted manually for pharyngeal pumping contractions over 15 s. Experiments were performed on 3 biological replicates with approximately 8 animals each, and statistics were calculated by an unpaired t-test using. GraphPad Prism 10 (Graphpad Software).

### Resistance to oxidative stress

1 mL of 250 mM Paraquat (Methyl viologen dichloride hydrate, 856177-1G, Sigma Aldrich) solution was added to a 60 mm NGM plates and plates were put on a shaker for 1 h at room temperature, followed by 1.5 h in a laminar flow hood to ensure plates were dry and the paraquat evenly distributed at room temperature. Animals were then transferred to paraquat plates at 20 °C and scored twice daily for survival by touch with a platinum wire. Animals that did not respond were scored as dead. Experiments were performed on 5 biological replicates with 25–30 animals each. Statistics were calculated by Kaplan–Meier survival curve utilizing a Log-rank (Mantel–Cox) test with Graphpad Prism 10 (Graphpad Software).

### Resistance to heat stress

*C. elegans* were transferred to 37 °C for either 2, 4, 6, 8, 12, or 16 h and scored for survival by touch with a platinum wire, and those that did not respond were scored as dead. Percent survival was calculated for each time point. Experiments were performed on 2–3 biological replicates with approximately 35 animals each, and statistics were calculated by Kaplan–Meier survival curve using a Log-rank (Mantel–Cox) test with Graphpad Prism 10 (Graphpad Software).

### Bacterial density (CFU)

*C. elegans* intestinal bacterial density measurement was done following^43^. Animals were counted and picked off plates into a 1.5 ml centrifuge tube containing M9 buffer, washed 3× with M9 buffer and then immobilized 0.25 mM levamisole and immersed into a 3% bleach solution to sterilize the exterior of the animal for 5 min. Animals were then washed 3× with M9 buffer, resuspended in 1% Triton X-100, then ground with a mini-pellet pestle. The samples were centrifuged at 14,000× *g* for 10 min at 4 °C then the supernatant removed, and the pellet was resuspended in 500uL M9 buffer. A series of tenfold dilutions were then made using ddH_2_O from 10^−1^ to 10^−5^ dilution. In a laminar flow hood, for each sample in triplicate, 50uL was pipetted onto a 35 mM LB agar plate and spread evenly across the surface. Plates were then air dried, and moved to 37 °C for 16 h, followed by colony counting. Only plates with colony counts between 30–300 were included, then they were multiplied by the dilution factor and divided by number of animals used to produce CFU/animal. Experiments were performed on 3 biological replicates with approximately 50 animals each, and statistics were calculated by an unpaired t-test with Graphpad Prism 10 (Graphpad Software).

### ROS quantification

Measurement of *C. elegans* reactive oxygen species (ROS) was done following^44^. *C. elegans* were picked off plates into a 1.5 ml centrifuge tube containing M9 buffer and washed 3× with M9 buffer. At the last wash, the animal mix was left in 100uL which was evenly distributed into two wells of a 96-well plate. Either 50uL M9 buffer (control) or 50uL of 50uM H_2_DCFDA was added to the well, then the plate was wrapped with aluminum foil and placed on a shaker for 30 min. A fluorometer was then used to measure fluorescence at 490 nm and 520 nm. Fluorescence of the blank control was subtracted from all samples and the relative difference in fluorescence calculated. Experiments were performed on 3 or more biological replicates with approximately 100 animals each, and statistics were calculated by an unpaired t-test with Graphpad Prism 10 (Graphpad Software).

### Fluorescent imaging and quantification

Transgenic *C. elegans* were mounted onto slides and imaged using a Hamamatsu ORCA ER camera mounted onto a Zeiss Axioskop 2 plus equipped with a FITC filter. Photographs were exported as .tiff files and quantified using ImageJ 1.52a (https://imagej.nih.gov/ij/index.html) by closely outlining whole individual animals and measuring pixel intensity. Images were captured in monochrome and artificially recolored as green using ImageJ. Experiments were performed on 2 or more biological replicates with approximately 10 animals per treatment, and statistics were calculated by an unpaired t-test with Graphpad Prism 10 (Graphpad Software).

### RNA extraction and RT‑qPCR

Animals were washed off plates with M9 buffer and rinsed twice with ddH_2_O. Total RNA was isolated using TRIzol Reagent with the Direct-zol RNA MiniPrep (Zymo Research). Quality control of the RNA was performed using a Nanodrop (Thermo Scientific) and samples with both an A260/A280 ratio > 2.0 and an A260/A230 ratio > 1.8 were used. First-strand cDNA synthesis was performed on 1.0 μg of total RNA using dNTPs, Oligo(dT)_12–18_ and SuperScript III Reverse Transcriptase (Invitrogen). Reverse Transcriptase Quantitative PCR was done using the QuantStudio™ 3 System (Applied Biosystems) with Power SYBR Green PCR Master Mix (Applied Biosystems) per the manufacturer’s instructions, in triplicate. The endogenous control for relative expression normalization was *act-1* for Figs. 1h,i, and 2b, and then *snb-1* for innate immune response genes in Fig. 3f. Sequences of primers can be found in Supplementary Table S2. Relative fold changes of gene expression were calculated using Comparative C_T_ (ΔΔC_T_). Experiments were performed on 3 or more biological replicates with approximately 100 animals each in triplicate, and statistics were calculated by an unpaired t-test with Graphpad Prism 10 (Graphpad Software).

### Intestinal distention

ERT60 jyIs13 [*act-5p::GFP::ACT-5* + *rol-6(su1006)*] L4s were picked onto control or glucose-fed *E. coli* plates and grown at 20 °C for 1, 6, or 12 days. Then, animals were mounted on slides and observed on a Zeiss Axioskop 2 plus for bacterial distention of the upper intestine. Distention was classified into three different levels: low, medium, and high. Distention level percentages were calculated for each replicate and averaged across all biological replicates. Experiments were performed on 3 or more biological replicates with approximately 10 animals per treatment, and statistics were calculated by an unpaired t-test with Graphpad Prism 10 (Graphpad Software).

### Dye uptake “Smurf” assay

Assay was adapted from^29^. *C. elegans* L4s were aged on control or glucose-fed *E. coli* for 0, 1, 3, 5, 6, 7, 9, 10, 12, or 14 days at 20 °C. Then, animals were picked off plates into an Eppendorf vial with 5% Erioglaucine disodium salt solution added and incubated at RT on a shaker plate for 3 h. Vials were centrifuged gently, and supernatant removed to 50uL. Using a glass pipet, animals were dispensed onto an OP50 NGM plate for 30 min. Animals that did not move or remained in the dye were omitted from further analysis. Then, animals were mounted onto slides and were imaged using a Zeiss AxioCam ERc 5 s mounted on a Zeiss Stemi SV11 Apo using bright field light. Experiments were performed on 2–3 biological replicates with approximately 10 animals per treatment and time point, and statistics were calculated by an unpaired t-test with Graphpad Prism 10 (Graphpad Software).

### Pathogen survival assay

*C. elegans* L4s were picked onto control or glucose-fed *E. coli* plates and aged for 1, 6, or 12 days at 20 °C. Animals were then picked onto either *P. aeruginosa*, *S. aureus*, or *E. faecalis* plates and scored 1–2 times per day for survival. Animals were scored by gently tapping with a platinum wire pick, those that did not respond were scored as dead. Animals that were lost, bagged, or died from vulva bursting were censored from the analysis. Experiments were performed on 2 biological replicates in triplicate, and statistics were calculated by a Kaplan–Meier survival curve utilizing a Log-rank (Mantel–Cox) test with Graphpad Prism 10 (Graphpad Software). Statistics of each experiment are located in Supplementary Table 1.

*P. aeruginosa* assays were performed in the laboratory of Dr. Read Pukkila-Worley. Briefly, plates were prepared using the protocol in^34,45^. A single colony of *P. aeruginosa* PA14 was inoculated into 3-mL of Luria–Bertani (LB) media and incubated at 37 °C for 14–15 h. 10 mL of this culture was added to 35-mm tissue culture plates containing 4 mL of slow kill agar. Plates were incubated for 24 h at 37 °C and 24 h at 25 °C and 0.1 mg/mL 5-fluorodeoxyuridine (FUDR) was added to the media 1–2 h prior to the start of the assay to prevent progeny from hatching. The *P. aeruginosa* survival assays were conducted at 25 °C.

*S. aureus* assays were performed in the laboratory of Dr. Javier Irazoqui. *S. aureus* plates were prepared using the protocol described in^46^. *S. aureus* SH1000 was grown overnight in TSB containing 50 mg/ml Kanamycin. Then 500–1000 ml of overnight culture was uniformly spread on the entire surface of freshly prepared 100 mm TSA plates supplemented with 10 mg/ml Kanamycin. The plates were incubated for 6 h at 37 °C, then stored overnight at 4 °C. The plates were warmed to room temperature and *S. aureus* survival assays were conducted at 20 °C. Scoring of the added glucose was done blind.

*E. faecalis* plates were prepared using similar methods as standard stock OP50 plates. Stock *E. faecalis* isolated from OMC47 human donors was inoculated into 20 mL LB and cultured for 16 h at 37 °C in an aerobic environment. Approximately 300 mL of the culture was aliquoted onto standard NGM plates, and then the plates were placed into an anaerobic chamber for 16 h at 37 °C. Plates were then kept at 20 °C and *C. elegans* were added.

### Supplementary Information

Supplementary Information.

## Data Availability

Data is provided within the manuscript and supplementary information files.
