# Supplementary material for: Glucose-fed microbiota alters C. elegans intestinal epithelium and increases susceptibility to multiple bacterial pathogens
Source: Sci Rep. 2024 Jun 7;14:13177. doi: 10.1038/s41598-024-63514-w (PMC11161463; doi:10.1038/s41598-024-63514-w)
Supplement: Supplementary file 1 — Supplementary Information. [file 41598_2024_63514_MOESM1_ESM.pdf]

Glucose fed microbiota alters *C. elegans* intestinal epithelium and increases susceptibility to bacterial pathogens

Samuel F. Kingsley[1], Yonghak Seo[1], Alicia Wood[1], Khursheed A. Wani[2], Xavier Gonzalez[2], Javier Irazoqui[2], Steven E. Finkel[3], and Heidi A. Tissenbaum[1,4,*]

[1]Department of Molecular, Cell and Cancer Biology, UMass Chan Medical School, Worcester, MA 01605, USA

[2]Department of Microbiology and Physiological Systems, UMass Chan Medical School, Worcester, MA 01605, USA

[3]Molecular and Computational Biology Section, Department of Biological Sciences, University of Southern California, Los Angeles, CA, 90089, USA

[4]Program in Molecular Medicine, UMass Chan Medical School, Worcester, MA, 01605, US

*Corresponding author: Heidi.Tissenbaum@umassmed.edu

Supplemental Figures 1-5

Supplemental Tables 1, 2

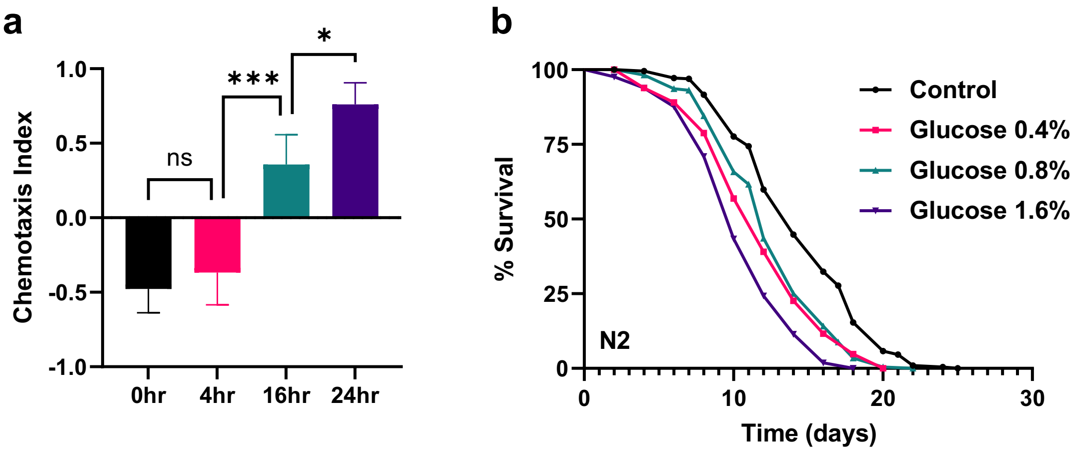

Supplementary Figure 1: Bacterial incubation with Glucose is required for changes in behavior (chemotaxis) and lifespan.

(a) Chemotaxis Index of *C. elegans* to *E. coli* incubated with glucose for 0, 4, 16, or 24 hrs, shown is mean ± s.d. (n= 412 total), ***$p<0.01$ comparing 4hr and 16hr, *$p<0.05$ comparing 16hr and 24hr by unpaired t-test). (b) Lifespan assay of *C. elegans* fed HT115 *E. coli* grown with 0, 0.4, 0.8, 1.6% glucose. Control mean ± s.d. = 13.6 ± 4.1 days (n= 287); Glucose 0.4% mean ± s.d. = 11.9 ± 4.0 days (n = 146) ***$p<0.01$ compared to control; Glucose 0.8% mean ± s.d. = 11.8 ± 3.7 days (n = 291) ****$p<0.01$ compared to control; Glucose 1.6%, mean ± s.d. = 10.6 ± 3.4 days (n = 210) ****$p<0.01$ compared to control by Log-rank (Mantel-Cox) test).

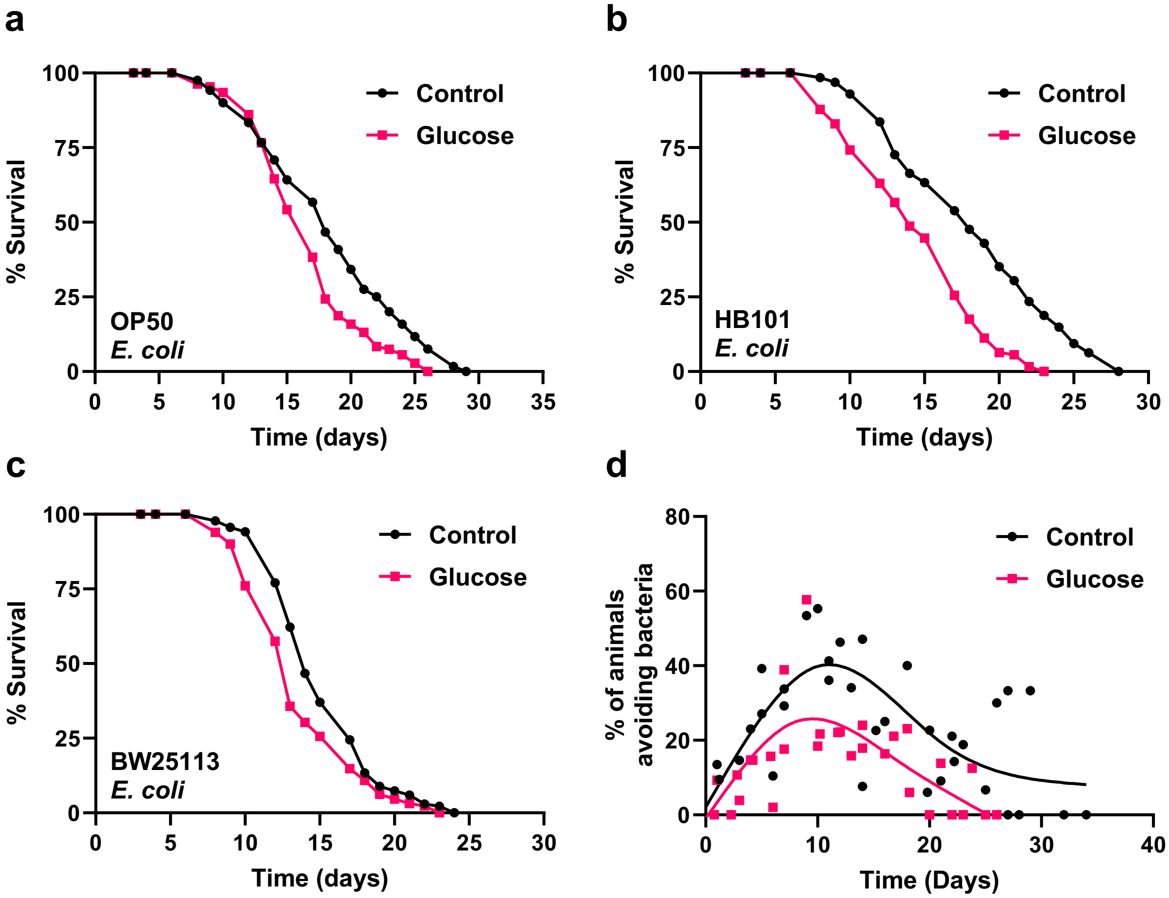

Supplementary Figure 2: Physiological consequences of a glucose fed microbiota

Lifespan effect of glucose fed microbiota across multiple strains of *E. coli.*
(a) Lifespan assay of *C. elegans* with glucose fed OP50 *E. coli* microbiota, Control mean ± s.d. = 18.3 ± 5.5 days (n = 120), Glucose mean ± s.d. = 16.5 ± 4.1 days (n = 107), ***p<0.001 by Log-rank (Mantel-Cox) test). (b) Lifespan assay of *C. elegans* with glucose fed HB101 *E. coli* microbiota, Control mean ± s.d. = 18.2 ± 5.3 days (n= 128), Glucose mean ± s.d. = 14.4 ± 4.3 days (n = 126), ****p<0.001 by Log-rank (Mantel-Cox) test). (c) Lifespan assay of *C. elegans* with glucose fed BW25113 *E. coli* microbiota, Control mean ± s.d. = 15.1 ± 3.4 days (n = 135), Glucose mean ± s.d. = 13.5 ± 3.6 days (n = 129), **p<0.01 by Log-rank (Mantel-Cox) test). (d) Avoidance behavior of *C. elegans* on glucose fed HT115 *E. coli* plates throughout the lifespan, measured as % of animals off bacterial lawn out of total animals per plate (n
= 490). Each dot represents a batch of approximately 30 animals, non-linear regression curve fit to data to show trend).

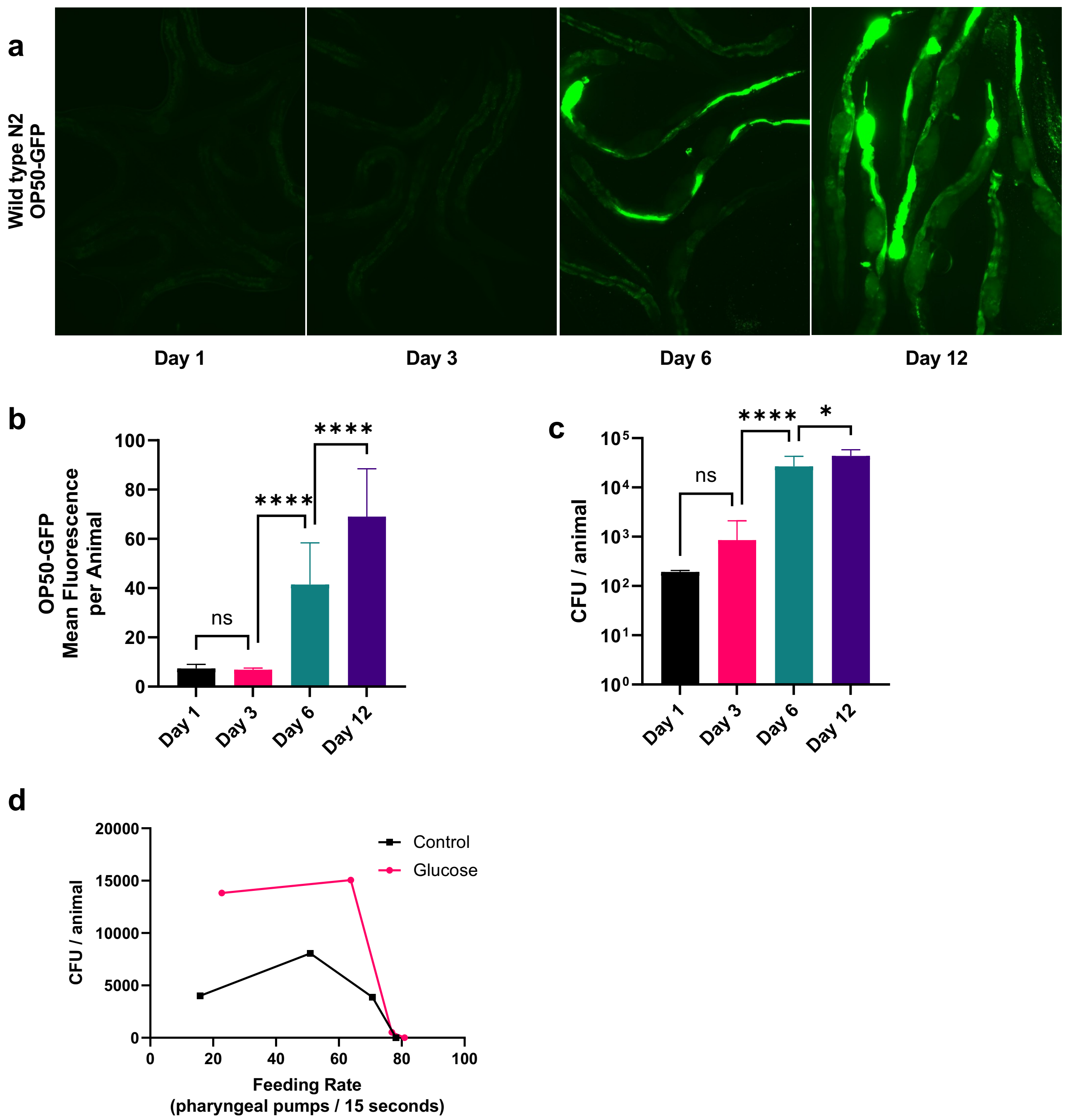

Supplementary Figure 3: Consequences of ingesting high glucose to *C. elegans and E. coli*

Growth of bacteria within *C. elegans*. (a) Representative images of OP50-GFP *E. coli* within the intestine of *C. elegans* after incubation for 1, 3, 6, or 12 days. (b) Quantification of OP50-GFP *E.*
coli fluorescence within the intestine after incubation for 1, 3, 6, or 12 days (mean ± s.d. (n = 76 total), not significant (ns) p>0.05 comparing day 1 and day 3, ****p<0.001 comparing day 3 and day 6, ****p<0.0001 comparing day 6 and 12 by unpaired t-test). (c) Bacterial density of OP50-GFP *E. coli* isolated from *C. elegans* after feeding for 1, 3, 6, or 12 days (mean ± s.d. (n = 700 total), not significant (ns) comparing day 1 and day 3, ****p<0.0001 comparing day 3 and day 6, *p<0.05 comparing day 6 and day 12 by unpaired t-test). (d) Correlation analysis of the mean bacterial density and mean pharyngeal pumping of age matched animals after growing with a control or glucose fed microbiota for 1, 3, 6, or 12 days (data from main Figures 1e and 2a).

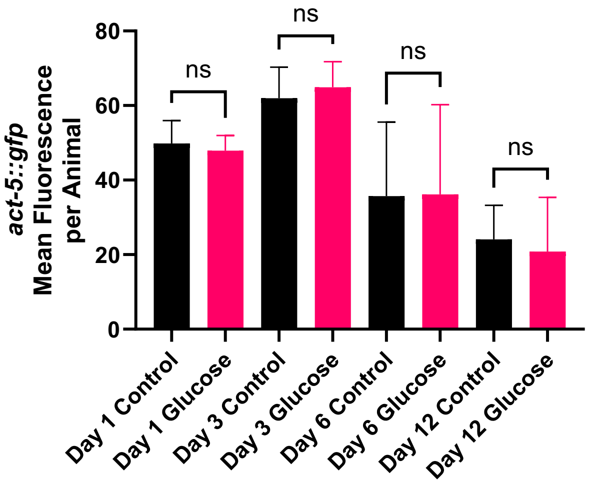

Supplementary Figure 4: A glucose fed microbiota results in changes in the intestinal epithelial structure.

Quantification of *act-5::gfp* whole body fluorescence after growing with a control or glucose fed microbiota for 1, 3, 6, or 12 days (mean ± s.d. (n = 243 animals total), not significant (ns) comparing age matched points by unpaired t-test).

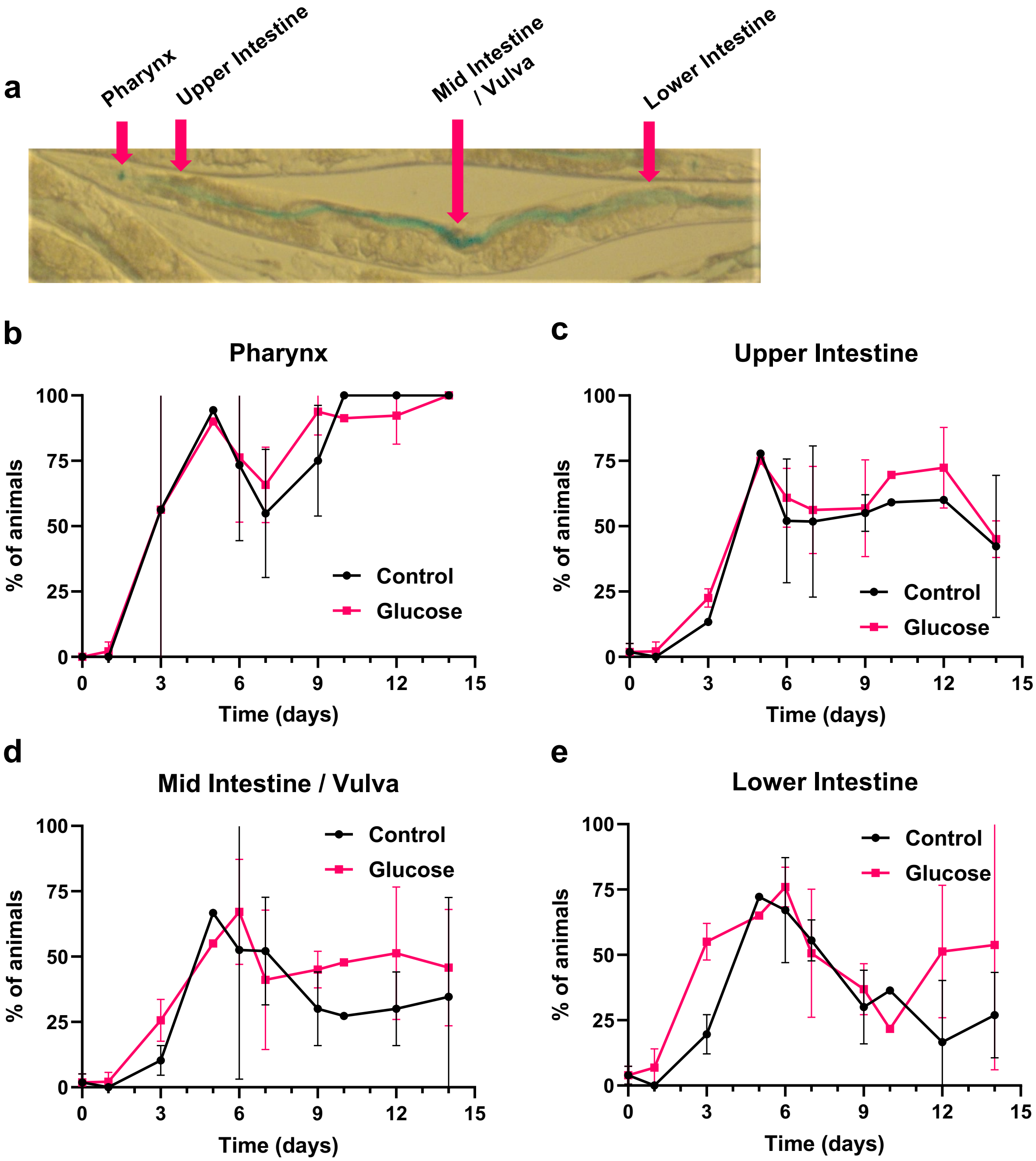

Supplementary Figure 5: Dye retention of animals with a glucose-fed microbiota

(a) Representative image of *C. elegans* stained with erioglaucine disodium salt showing dye retention in the pharynx, upper intestine, mid intestine/vulva, and lower intestine. (b) Quantification of erioglaucine disodium salt dye retention in the pharynx of *C. elegans*, each dot represents 10-25 animals. (c) Quantification of erioglaucine disodium salt dye retention in the upper intestine of *C. elegans*, each dot represents 10-25 animals. (d) Quantification of erioglaucine disodium salt dye retention in the mid intestine/vulva of *C. elegans*, each dot represents 10-25 animals. (e) Quantification of erioglaucine disodium salt dye retention in the lower intestine of *C. elegans*, each dot represents 10-25 animals. (b-c Control n = 283, Glucose n = 281 animals, each data point is a batched sample of 10-20 animals)

Table S1: Lifespan Statistics

Figure	Strain	Bacteria / Treatment	Mean Lifespan (Days)	Standard Deviation (+/- Days)	number of animals (n)	*p* value
1c	N2	0% control fed HT115	16.5	4.82	1028	-
1c	N2	0.8% glucose fed HT115	14.2	4.27	1049	<0.0001
1f	N2	0% control fed HT115 then 37°C heat	0.259	0.0546	347	
1f	N2	0.8% glucose fed HT115 then 37°C heat	0.207	0.025	344	<0.0001
1g	N2	0% control fed HT115 then Paraquat	1.21	0.667	101	
1g	N2	0.8% glucose fed HT115 then Paraquat	1.3	0.663	142	0.1081(ns)
4a	N2	1 day aged before *P. aeruginosa*	3.72	1.12	47	-
4a	N2	6 day aged before *P. aeruginosa*	2.98	1.16	100	0.0014
4a	N2	12 day aged before *P. aeruginosa*	2.3	1.17	44	<0.0001
4b	N2	0% control fed HT115 then *P. aeruginosa*	2.98	1.16	100	-
4b	N2	0.8% glucose fed HT115 then *P. aeruginosa*	2.5	0.994	107	0.0019
4c	N2	0% control fed HT115 then *S. aureus*	1.55	0.629	230	-
4c	N2	0.8% glucose fed HT115 then *S. aureus*	1.2	0.471	275	<0.0001
4d	N2	0% control fed HT115 then *E. faecalis*	10.3	3.86	64	-
4d	N2	0.8% glucose fed HT115 then *E. faecalis*	7.98	4.7	62	0.0147
Supp 1c	N2	0% control fed HT115	13.6	4.08	287	-
Supp 1c	N2	0.4% glucose fed HT115	11.9	4.03	146	0.0001
Supp 1c	N2	0.8% glucose fed HT115	11.8	3.65	291	<0.0001
Supp 1c	N2	1.6% glucose fed HT115	10.6	3.42	210	<0.0001
Supp 2a	N2	0% control fed OP50	18.3	5.51	120	-
Supp 2a	N2	1% glucose fed OP50	16.5	4.11	107	0.0004
Supp 2b	N2	0% control fed HB101	18.2	5.33	128	-
Supp 2b	N2	1% glucose fed HB101	14.4	4.26	126	<0.0001
Supp 2c	N2	0% control fed BW25113	15.1	3.39	135	-
Supp 2c	N2	1% glucose fed BW25113	13.5	3.57	129	0.0021

Statistics calculated against appropriate control using a Kaplan-Meier survival curve and Log-rank (Mantel-Cox) test

Table 2: RTqPCR primer pair sequences

Gene Amplified	**Sequence (5'-3')**	**Source**
act-1 (control)	CTCTTGCCCCATCAACCATG	Kwon et al 2010
act-1 (control)	CTTGCTTGGAGATCCACATC	Kwon et al 2010
act-5	CCAATCTATGAAGGATATGCCC	GETprime Ensembl release 81
act-5	CATCATGTAGTCGGTCAAGTC	GETprime Ensembl release 81
clec-7	GGCCGGCTTCAAATGTTTATC	Kumar et al., 2019
clec-7	TAGTGGACATTACCATGCAGTC	Kumar et al., 2019
clec-60	CTGAGCCAAGAACCACAAGA	Kumar et al., 2019
clec-60	GAAGTGCTGACTGACGAAAGA	Kumar et al., 2019
clec-67	TGATGGTGACAGTTCAAAGC	Pukkila-Worley et al 2012
clec-67	TTCCAAAAATGCCCGAGTAG	Pukkila-Worley et al 2012
clec-82	TTCCGCCGTTGTCTGTTT	Kumar et al., 2019
clec-82	CACTTGAGCTGGCTAGATTGA	Kumar et al., 2019
C17H12.8	TGTCATTTCAATGGAGGATATTGT	Cheesman et al 2016
C17H12.8	TGATGGAGTTGGAGGATATTGA	Cheesman et al 2016
F53A9.8	GTTCACCATGCAGGAGATCA	Kumar et al., 2019
F53A9.8	TCTCCATCTTGGTGTTGAGTTT	Kumar et al., 2019
gst-4	GCTGAAGCCAACGACTCCAT	Park, S-K. et al 2009
gst-4	GACCGAATTGTTCTCCATCGA	Park, S-K. et al 2009
hlh-30	GAACACATCAGAAGACATGAAAC	Wani et al 2021
hlh-30	AAGATGCGATGGCGGGACCT	Wani et al 2021
hsf-1	TCAGACAGTTGAATATGTACGG	GETprime Ensembl release 81
hsf-1	CCTGATCTGATTCTGTTCGAG	GETprime Ensembl release 81
ifb-1	CTCGTAGAAAGGCAGAGGT	GETprime Ensembl release 81
ifb-1	TATCTTCGTATCTGGATCTGTACTC	GETprime Ensembl release 81
ifb-2	CTACAGACACAGATATGAAGAGG	GETprime Ensembl release 81
ifb-2	TGTGCTTGTGAAATAGCTCC	GETprime Ensembl release 81
irg-4	CACAATGATTTCAATGCGAGA	Pukkila-Worley et al 2012
irg-4	TGCTTTCAGAACACAGTCAGG	Pukkila-Worley et al 2012
irg-5	TTCAAGAATTGTCCCCAACA	Pukkila-Worley et al 2012
irg-5	TGTGGAAGTTGGTTGTTTCC	Pukkila-Worley et al 2012
irg-6	AAATCTATCACCTGGATCACGA	Pukkila-Worley et al 2012
irg-6	CCTTGATATTTCGTCCATCG	Pukkila-Worley et al 2012
lys-1	CTGGATTCAGGTTACCTCCC	GETprime Ensembl release 81
lys-1	CGTGAAATAATGCTGTTGAGGA	GETprime Ensembl release 81
lys-5	CGGGAAGTGTAGATACTGTTGG	Kumar et al., 2019
lys-5	AGAGACGCCTTAACTTGGTTAG	Kumar et al., 2019
lys-7	GCCGTCAAACTTGGCATCTT	Kwon et al 2010
lys-7	GGGTTGTATGCACGAACGAA	Kwon et al 2010
lys-8	GTGTCAAGAGCTAGACAATATGG	GETprime Ensembl release 81
lys-8	TCCATCCGTTGGTGATCTG	GETprime Ensembl release 81
mul-1	CCATCAACTACGCCAAAGC	Pukkila-Worley et al 2012
mul-1	TCCGGTGGATAGAAGGTGTT	Pukkila-Worley et al 2012
pmk-1	ACTTCATCCGACTCCACGAG	Dinic et al 2021
pmk-1	CAGCAGCACAAACAGTTCCA	Dinic et al 2021

Table 2: RTqPCR primer pair sequences (continued)		
Gene Amplified	**Sequence (5'-3')**	**Source**
snb-1 (control)	CCGGATAAGACCATCTTGACG	Pukkila-Worley et al 2012
snb-1 (control)	GACGACTTCATCAACCTGAGC	Pukkila-Worley et al 2012
spp-1	TGATGTCTGCAAGGCTCTC	GETprime Ensembl release 81
spp-1	CATCAAGCCATGCATCGAG	GETprime Ensembl release 81
sysm-1	AGACCATCATGCCTTCACT	Cheesman et al 2016
sysm-1	GTAACGCAGACACCACAGGT	Cheesman et al 2016
